# Exercise interventions can improve muscle strength, endurance, and electrical activity of lumbar extensors in individuals with non-specific low back pain: a systematic review with meta-analysis

**DOI:** 10.1038/s41598-021-96403-7

**Published:** 2021-08-19

**Authors:** Sacha Clael, Lorrane Freitas Campos, Karina Lisboa Correia, Joana Marcela Sales de Lucena, Paulo Gentil, João Luiz Durigan, Alexandre Lima de Araújo Ribeiro, Wagner Rodrigues Martins

**Affiliations:** 1 Faculty of Physical Education, University of Brasília, Brasília, DF Brazil; 2 Department of Physiotherapy, University of Brasília, Brasília, DF Brazil; 3 University Federal of Tocantins, Palmas, TO Brazil; 4 Faculty of Physical Education and Dance, Federal University of Goiás, Goiás Goiânia, Brazil

**Keywords:** Anatomy, Health care

## Abstract

Exercise interventions have been recommended for people with non-specific low back pain. The literature is scarce regarding the effects of exercise on muscle strength, endurance, and electrical activity of lumbar extensor muscles. Electronic searches were carried out from May 2020 until August 2020 in the following databases: PUBMED, CENTRAL, EMBASE, PEDro, SPORTDiscus, Scielo, and LILACS. Only randomized controlled trials with passive and active control groups were included. The methodological quality of the included studies was performed using the Physiotherapy Evidence Database Scale. Eight studies, involving 508 participants, were included in metanalytical procedures. Exercise interventions demonstrated superior effects on muscle activity (Electromyography) when compared with active controls (*p* < 0.0001). Exercise interventions demonstrated superior effects on muscle endurance (Sorensen Test) when compared with passive (*p* = 0.0340) and active controls (*p* = 0.0276). Exercise interventions demonstrated superior effects on muscle strength (Machine) when compared with passive controls (*p* = 0.0092). Exercise interventions can improve muscle strength, endurance, and electrical activity in people with non-specific low back pain.

## Introduction

Approximately 80% of adults experience lower back pain (LBP) at some time in their lives^1^. In 1990, for all ages and both sexes, the leading cause of years lived with disabilities was LBP (42.5 million years lived with disabilities)^2^. Between 1990 and 2007, the number of all-age years lived with disabilities attributed to LBP increased by 30%^2^. LBP leads the cause of years lived with disabilities in 126 of 195 countries according to the Global Burden of Disease Study from 2007 to 2017^2^. In 2019, for ages 50–74 years, LBP remained in the top-ten-ranking causes of years lived with disabilities^3^.

LBP has a strong socioeconomic impact^4^, which leads to a decrease in people’s quality of life and productivity^5^, and an increase in direct and indirect costs with palliative treatments^6^. The annual cost of a person with LBP is approximately 7000 euros^7^. When added to the unproductive occupational behavior, the costs rise to approximately 18,000 euros^7^. In Brazil, the annual loss of productivity per individual with LBP costs approximately 2684 Dollars^8^, and in the US the annual loss of productivity cost per individual is approximately 1685 Dollars^9^. LBP also affects employment in the informal sector^10^, which raises the hypothesis that the data mentioned above could be higher.

Approximately 90% of LBP patients do not present a pathoanatomical diagnosis, so are frequently diagnosed with non-specific low back pain (NSLBP)^11^. Therefore, in NSLBP, the symptoms are not associated with specific tissue damage causes^12^. One of the main treatments of NSLBP is exercise, such as exercise therapy^13^, Pilates^14^, motor control exercises^15^, and back schools^16^. Considering the exponential growth of randomized clinical trials investigating the efficacy of exercise interventions in individuals with NSLBP, in recent decades, many systematic reviews have explored the association between exercise and back pain outcomes.

The major recommendations of these systematic reviews are based on patient-centered outcomes, such as pain intensity, disability, and global perception of recovery. The classic examples are Cochrane reviews of exercise therapy^13^, back schools^16^, Pilates^14^, and motor control exercises^15^. Considering that almost all review papers provide information only about subjective measures, there is a lack of evidence on objective measures in routine settings, such as muscle strength and muscle endurance.

Regarding these objective outcomes in review articles, to our knowledge, only two studies investigated the specificity of exercises (isolated lumbar extension resistance training) to improve lumbar extensor outcomes, such as muscle strength and endurance in individuals with back pain^17,18^. The results show that isolated exercises for lumbar extensors can produce chronic muscle adaptation^17^. Although the studies of Steele et al.^17,18^ provided some evidence about the association of a specific exercise intervention for improvements in muscle strength and endurance of lumbar extensors, this was not a systematic review, and no meta-analytical procedures were employed. Therefore, insights about specific exercise approaches in people with NSLBP are needed. Considering this gap in the literature of evidence about the efficacy of exercise interventions on objective outcomes of muscle functions, a new systematic review with meta-analysis would be useful. Therefore, the present study aimed to compare exercises for increasing trunk extensor muscle function with exercises that are not explicitly designed to increase muscle strength in people with NSLBP, for the following outcomes: electromyography (EMG), and muscle strength and endurance.

## Methods

### Preliminary setting

This study was registered in the International Prospective Register of Systematic Reviews (CRD42020188914; http://www.crd.york.ac.uk/PROSPERO/) and was reported according to the Preferred Reporting Items for Systematic Reviews and Meta-analysis (PRISMA; http://www.prisma-statement.org)^19^.

The study was designed according to the following PICO strategy^20^: adults 18 to 55 years of age (Population), exercise interventions (Intervention), other interventions, whether active or passive (Comparison), strength, endurance, and/or muscle activity (Outcomes).

### Inclusion criteria

#### Types of studies

We included only randomized controlled trial studies^21^ performed for more than 6 weeks. In the preliminary searches, a sufficient number of randomized controlled trials were found to justify this criteria design for eligibility and answer the study question^22^. Therefore, we decided not to include non-randomized controlled trials^22^. Regarding the six week criteria, this period was chosen based on previous literature, according to the initial phase of neuromuscular adaptations from resistance training^23^.

#### Type of participants

We included studies with any type of NSLBP (acute, sub-acute, chronic) in adult individuals, aged between 18 and 55 years, with no restriction on sex. LBP was defined as pain and or discomfort located below the ribs and above the gluteal crease^24^. NSLBP is not attributed to a recognizable or specific pathology^25^ and we considered for this study LBP with or without referred leg pain. We excluded studies with participants that had undergone spine surgery, osteoporosis, fractures, and malignancies. Patients with systematic diseases or non-mechanical LBP (e.g., disc herniation, spinal stenosis, etc.), who were pregnant, experienced postnatal-related LBP, and military forces were excluded.

#### Type of interventions

We included studies comparing an experimental group (exercise interventions for increasing trunk extensor muscle function) versus passive and or active controls. For passive controls, we considered: no intervention and waiting list groups. For active groups, we considered: standard care (e.g., multimodal physical therapy) and different types of exercise that are not explicitly designed to increase muscle strength, such as aerobic exercise, Yoga, stretching exercises, home-based exercises, circuit-based exercises, telerehabilitation, and Tai Chi Chuan. We followed the Cochrane Handbook for Systematic Reviews of Intervention to define the control group classifications^22^. When the experimental group was used in addition to another active treatment, the trial was included (e.g. [exercise intervention plus stretching] versus [stretching]).

We excluded studies that compared two different types of exercise interventions for increasing muscle strength, endurance, or electrical activity of trunk extensors (e.g., motor control exercise versus machine strength exercise). This decision was made considering that there is no standard gold method of exercise for the treatment of LBP patients^15^ and the present study was not developed to investigate the best exercise (comparisons between exercises designed to increase muscle strength for example).

We decided to cluster the analysis of interventions (different exercises), considering that they fit the definition of physical training, in which the muscle moves or tries to move against an opposing force. In the case of isometric exercises, we considered that gravity is a force to be overcome^26^.

#### Types of outcome measures

Continuous data for meta-analysis were obtained from general outcomes designed to assess^21^ muscle strength, muscle endurance, and muscle activity of trunk extensor muscles^24^, such as EMG, and muscle strength measured using direct (e.g., isometric and dynamic dynamometers) and indirect (e.g., Biering–Sorensen test) methods.

To analyze muscle activity and fiber recruitment, surface electromyography equipment (time and frequency analysis) has been employed as the gold standard for many years to study normal and altered outcomes, such as maximal isometric muscle contraction, and the units are presented in Hertz^27^. The isokinetic dynamometer allows assessment of strength during a dynamic or isometric contraction. The dynamometer resistance is equal to the muscular forces applied to the machine, and the units presented are in Newton-metres^28^. The Sorensen test measures the amount of time a person can hold the unsupported upper body in a horizontal prone position with the lower body fixed on the examining table. The units presented are in s^29^.

### Search methods for identification of studies

Electronic searches started in May 2020 and were conducted in the following databases until August 2020: PUBMED, CENTRAL, EMBASE, PEDro, SPORTDiscus, Scielo, LILACS. Only articles written in English were included, but there were no restrictions imposed on the publication date. The “ClinicalTrials.gov” database was used to identify potential unpublished studies and ongoing studies. Google Scholar was used to assess the grey-literature (thesis, clinical report, conference abstract).

Research strategies were conducted and designed depending on the specific settings of each database. A dedicated search strategy was prepared for each database. According to the PICO model of a clinical question (only participants and interventions), MeSH (Medical Subject Headings) terms and text words (e.g., low back pain; exercise; strength training) were used and combined with Boolean operators (AND, OR). Additionally, a manual search was conducted through the bibliographies of all included studies to obtain an integrative cross-referenced full-text selection. We report the primary core search strategy used in the databases consulted (Supplementary Material, Supplementary Table S1). In addition, Endnote version 8.0 was used to assess duplicated references from the database searches.

### Data collection and analysis

#### Selection of studies

Two review authors (SC, LFC) independently screened all titles and abstracts retrieved by the search strategy for eligibility. Those deemed potentially relevant were retrieved for full-text assessment by the same authors (SC, LFC), who assessed whether the reports fulfilled the selection criteria. When necessary, a third review author (WRM) resolved any disagreements regarding study inclusion. We used a PRISMA flowchart to summarize the search results and the study selection process^30^.

#### Data extraction and management

Two review authors (SC, LFC) independently extracted the primary data from the studies using a standard data extraction form on Excel software to collect the following details: participants, intervention, comparator, outcomes, assessment, conclusion, and financial support (Table 1). In addition, participants, intervention, comparator, and outcomes were extracted, as shown in Table 2. The extraction was checked by a third reviewer (ALAR).

**Table 1:** Characteristics of included studies (*n* = 17).

Year, title	Participants	Age	Duration	Intervention	Comparator	Assessment	Outcomes	Follow-up	Financial support
Chok, 1999	*Sex* Male and Female *Classification* Subacute *Radiation* With or without	*Exercise* Mean: 37.5 SD: 9.7 *Control* Mean:34.2 SD: 8.1	6 weeks (3 times a week)	Exercise (*n* = 30) 4 Levels of shoulder lift exercises (30–45 min)	No intervention (*n* = 24) Postural and back care advice and the back-care booklet	Sorensen test (s)	↔ Endurance of trunk extensors	No	No information available
Mannion, 2001*	*Sex* Male and Female *Classification* Chronic *Radiation* Without	Age Mean: 45.0 SD: 10.0	12 weeks (2 times a week)	Training devices (*n* = 41) 3 planes Submaximal isoinertial—25 repetitions/session on each device—1 h	Physiotherapy + home exercises (*n* = 46) Isometric and therabands—30 min Aerobic/stretching (*n* = 45)—1 h	Sorensen test (s) Electromyogram (Hz)	↑ Isometric strength < devices group ↑ Activation of the erector spinae extension ↑ Endurance	No	No information available
Rittweger, 2002	*Sex* Male and Female Classification Chronic *Radiation* Without	*Isodynamic* Mean: 49.8 SD: 6.6 *Vibration* Mean: 54.1 SD: 3.4	12 weeks (1 or 2 times a week)	Isodynamic lumbar extension exercise (*n* = 25) Resistance exercise of the abdominal and thigh muscles	Whole-body vibration exercise (*n* = 25) A platform that oscillates between the subject’s feet	LE Mark1 Lumbar extension machine (Nm/kg)	↑Lumbar extension torque < Isodynamic lumbar extension exercise group	6 months	No information available
Maul, 2005	*Sex* Male and Female *Classification* Chronic *Radiation* Without	*Exercise* Mean: 38 SD: 8 *Comparison* Mean: 39 SD: 10	Exercise: 12 weeks (2 times a week) Comparison: 3 sessions	Exercise group strengthening exercises + back school (*n* = 74) Static and dynamic exercises with small weights, machines	Comparison Low Back School (*n* = 74) Informational classes	Sorensen test (modified) (s) Isokinetic dynamometer (Nm)	Both groups < in the exercise group: ↑ Muscular endurance ↑Isokinetic strength	1 year and 10 years	No information available
Harts, 2008*	*Sex* Male *Classification* Chronic *Radiation* With or without	HIT Mean: 44 SD: 10 LIT Mean: 42 SD: 10 Control Mean: 41 SD: 9	8 weeks (1 to 2 times a week)	High-intensity training- HIT (*n* = 20) 1 Progressive resistance exercise program for the isolated lumbar extensor muscles	Low-intensity training (*n* = 19) One non-progressive, low-intensity resistance No intervention Control (*n* = 21) Waiting list	Modified lower back machine (Nm)	↑Isometric back strength ↔ Between HIT and LIT	24 weeks	No information available
Kell, 2009*	*Sex* Nod reported *Classification* Chronic *Radiation* Without	Resistance Mean: 40.1 SD: 8.7 Aerobic Mean: 36.7 SD: 8.9 Control Mean: 35.3 SD: 7.3	16 weeks (3 times a week)	Resistance exercises (*n* = 9) 12 Upper-and lower-body RT exercises that consisted of free weights, machines, and body weight	Aerobic training (*n* = 9) elliptical and treadmill No intervention control (*n* = 9)	Sorensen test 10RM testing	Resistance group: ↑Musculoskeletal fitness	No	Yes
Macedo, 2010	*Sex* Female *Classification* Chronic *Radiation* Without	Isostretching. group Mean: 21.11 SD: 2.02 Control Mean: 20.6 SD: 0.81	20 sessions (3 times a week)	Isostretching (*n* = 9) 9 Isometric contractions of glutes, quadriceps, abdominals, and pelvic floor	No intervention control (*n* = 6)	Test of maximum repetition in one minute (RM)	Isostretching: ↑ Muscular resistance abdominals, gluteus maximus, and trunk extensors	No	No information available
Bronfort, 2011	*Sex* Male and Female *Classification* Subacute *Radiation* with or without	Supervised Mean:44.5 SD: 11.8 Home Mean: 45.6 SD: 10.3 Chiropractic Mean: 45.2 SD: 10.8	12 weeks (2 times a week)	Supervised Exercise Therapy (*n* = 100) Seven exercises focused on the low back and abdominal musculature + core strengthening	Home exercise (*n* = 101) Three strengthening + advice and stretching Chiropractic Spinal Manipulation (*n* = 100) low back and sacroiliac	Sorensen test Lumbar dynamic motion (Orthopedic Systems)	Supervised > Chiropractic and Home exercise: ↑ Muscle endurance ↑ Strength	52 weeks	No information available
Smith, 2011	*Sex* Not reported *Classification* Chronic *Radiation* Without	Age Mean: 42.93 SD: 10.80	12 weeks (1 time a week)	Lumbar extension training with pelvic stabilization (STAB) (*n* = 15)	Lumbar extension training without stabilization (*n* = 15) No intervention (*n* = 12)	Lumbar Extension Machine (Nm)	STAB group: ↑ Lumbar strength at all joint angles	No	No information available
França, 2012	*Sex* Not reported *Classification* Chronic *Radiation* Not reported	Stretching Mean: 41.53 SD: 4.41 Seg. Stab. Mean: 42.07 SD: 8.15	Six weeks (2 times a week)	Stretching (*n* = 15) 4 Exercises focused on stretching the erector spinae, hamstrings, and triceps surae	Segmental stabilization (*n* = 15) 4 Exercises focused on the TrA and lumbar multifidus muscles	Pressure Biofeedback Unit (mmHg)	Both treatments: ↑ Transversus Abdominis Activation Capacity	No	No information available
Bruce-Low, 2012*	*Sex* Not reported *Classification* Chronic *Radiation* Without	Age Mean: 45.5 SD: 14.1	12 weeks (2 or 1 time a week)	Exercise (*n* = 20) (twice a week) 1 Lumbar extension machine 8–12 rep—80% of the maximum TFT	Exercise (once a week) (*n* = 31) 1 Lumbar extension machine No intervention (*n* = 21)	Lumbar extension machine (Dynamometer) (Nm)	Both training: ↑ Maximal strength ↑ Range of motion and	No	No information available
Alp, 2014	*Sex* Female *Classification* Chronic *Radiation* Without	Core exercise Mean: 48 SD: 33.74 Home-based Mean: 51 SD: 48.73	6 weeks (3 times a week)	Core stabilization exercise (*n* = 24) 4 Stretching, stabilization exercises for the multifidus/ transversus abdominis muscles	Home-based exercise (*n* = 24) 2 Lumbar isometric and lumbar flexion–extension exercises 20 rep	Sorensen test (s) Kraus-Weber test (sec)	Both treatments: ↑ Endurance abdominal muscles and dorsal extensors	No	No financial support
You, 2015	*Sex* Not reported *Classification* Chronic *Radiation* Without	Training Mean: 27.6 SD: 5.6 Control Mean: 27.6 SD: 6.7	6 weeks (2 times a week)	Training group: Exercise (*n* = 7) 6 Stabilization exercise using a S.E.T system	No intervention (*n* = 5)	Muscular strength dynamometer (kg-m / BW)	Training group: ↑Muscular strength ↑Endurance	No	Yes
Lomond, 2015	*Sex* Male and female *Classification* Chronic *Radiation* Without	Stabilization Mean: 43.1 SD: 11.9 Control Mean: 41.6 SD: 10.9	7 weeks	Trunk stabilization (*n* = 12) Motor control, strengthening, submaximal efforts STB and an education booklet	Movement system impairment (*n* = 21) Education booklet	Electromyogram (Hz)	APA characteristics (i.e., force application or EMG amplitude)	12 months	Yes
Knox, 2017	*Sex* Male and female *Classification* Chronic *Radiation* Without	Exercise Mean: 33.9 SD: 1.9 Control Mean: 34.6 SD: 2.2	8 weeks (3 times a week)	Exercise (*n* = 12) Pilates, 3 exercise sessions	No intervention control (*n* = 12)	Electromyogram (Hz)	↑ Ipsilateral transverse abdominis/internal oblique ↑Control of rotational torques	No	No information available
Cortell-Tormo, 2018*	*Sex* Female *Classification* Chronic *Radiation* Without	Exercise Mean: 35.6 SD: 7.9 Control Mean:35.6 SD: 9.7	12 weeks (2 times a week)	Exercise (*n* = 11) 18 Upper and lower body resistance training exercise—free weights, gym and body weight	No intervention control (*n* = 8)	Balance (trials to 60 s); Curl-up (rep); Squat (rep); Static back (s); Side bridge(s)	↑ Physical function ↑ Balance ↑ Squat ↑ Static back ↑ Side Bridge	No	Yes
Bello, 2018*	*Sex* Male and Female *Classification* Chronic *Radiation* Without	Stab. group Mean:42.2 SD: 12.91 Treadmill Mean: 46.6 SD: 11.6	8 weeks (3 times a week)	Stabilization exercises (*n* = 25) 4 exercises—McGil protocol	Treadmill walking exercise (*n* = 25) Modified Bruce protocol	Electromyogram (Hz)	↑ Multifidus muscle activation < Stabilization exercises	No	No information available

*HIT* high-intensity training, *LIT* low-intensity training, *TrA* transversus abdominis.

*Studies included in the meta-analysis.

↔ No significant difference, ↑ Significant Increase, ↓ Significant Decrease, < Significantly more.

**Table 2:** Ongoing studies identified on web-based protocol registers.

Year, title	Title	Participants	Intervention	Comparator	Outcomes
Bronfort, 2005	Chiropractic and exercise for seniors with low back pain	Adults more than 65 years old	Supervised rehabilitative exercise + home exercise	Chiropractic Manual treatment + home exercise Home exercise	Primary: patient-rated pain Secondary: general health, disability, Satisfaction, medication use, and biomechanical test: Lumbar spinal motion Trunk strength & Functional endurance Ability Observed Pain Behavior
França, 2010	Lumbar stabilization, strengthening and stretching in chronic low back pain	Adults from 23 to 53 years old	Strengthening	Stabilization group Stretching group	Primary: pain, functional disability, and TrA muscle activation capacity
Da Silva, 2014	Effect of volume training on back endurance	Adults from 18 to 35 years old	Three sets of exercise	One set of exercise No intervention	Primary: isometric endurance Secondary: number of repetitions Others: EMG fatigue index, Isometric strength
Pennone, 2017	Effect of strength training for chronic low back pain patients	Adults 18 years and older	Strength training	Usual care	Primary: low back pain intensity, Back pain recurrence Secondary: Roland-Morris disability, muscle endurance, use of analgesics, handgrip strength, pain sites
Martins, 2018	Efficacy of exercises in individuals with non-specific chronic low back pain	Adults from 18 to 50 years old	Resistance training	Motor Control	Primary: numerical rating scale, disability and kinesiophobia Secondary: trunk muscle strength
Simões, 2018	Exercise protocol for pilots with back pain	Adults from 25 to 45 years old	Exercise (*n* = 10)	No intervention (10)	Primary: change in number on Visual Analog Scale (VAS) of pain sensation in areas of the Body and disability Secondary: presence of injuries, postural pattern, musculoskeletal disorders, range of motion, and endurance

#### Methodological quality

Two review authors (KLC, ALAR) independently assessed the methodological quality of the included RCTs using the Physiotherapy Evidence Database Scale (PEDro) scores. The PEDro scale consists of 11 criteria: random allocation, concealed allocation, baseline comparability, blind subjects, blind therapists, blind assessors, adequate follow-up, intention-to-treat-analysis, between group comparisons, point estimates and variability). The items assessed receive either a “yes”, or “no” rating. The maximum PEDro score is 10 points. Trials with a PEDro score ≥ 6 points were classified as high-quality, while trials with a PEDro score of < 6 were classified as low-quality^31^. Any disagreement was resolved by a third review author (WRM).

#### Measures of treatment effects

Considering that the values of outcomes investigated were continuous variables and the scale of measurement, the mean differences (MD) and 95% confidence intervals (CIs) were used. The MD can be used as a summary statistic in a meta-analysis when all study outcome measurements are made on the same scale^22^. The MD is a standard statistic that measures the absolute difference between the mean values in the groups of a randomized trial. A common practical problem in the meta-analysis of change scores is when the study did not report the standard deviation (SD) of change scores; therefore, we decided to extract the data from post-intervention values (this assumption avoids the need to impute the SD of the changes)^22^. The post-intervention values for meta-analysis procedures were obtained using the first time point close to the end of the treatment because few studies reported follow up measurements. For statistical analysis, the continuous data were extracted to a database on Excel Software (Version 16.42) before using RStudio software (Version 1.4.1106, RStudio, Inc) with the following packages: “meta”, “metafor”, “readr”, “Rcpp”, “BH” and “readxl” to perform the appropriate metanalytical procedures.

#### Assessment of heterogeneity and sensitivity

The heterogeneity of the studies was assessed by the I^2^ statistic and 95% CI^32^. The following I^2^ statistics were considered: 0–40% might not be significant, 30–60% may represent moderate heterogeneity, 50–90% may represent substantial heterogeneity, and 75–100% may represent considerable heterogeneity^32^. Since the included studies have distinct populations, intervention parameters, and settings, a random-effect was always used. This decision was made based on the expectation that the intervention effects are not truly identical between studies. We decided not to choose between fixed-effects and random-effects according to the statistical test results for heterogeneity^22^. Considering that the variables used to perform the meta-analytical procedures were established clearly and a priori (eligibility criteria, continuous data [analysis on post-intervention], and analysis methods [random effects; mean difference dimension]), the sensitivity analysis was not employed considering these assumptions.

#### *Level *of confidence in meta-analytical results

The quality of the evidence was rated using the Grading of Recommendations, Assessment, Development, and Evaluation (GRADE). GRADE offers four levels of evidence: high, moderate, low, and very low. Randomized trials begin as high quality evidence, and the quality may be downgraded according to limitations in five domains: study design and risk of bias, inconsistency of results, indirectness of evidence, imprecision, and other (for example, publication bias). If there were sufficient data available to use quantitative analysis for summarising the data, we assessed the quality of the evidence for each outcome. To summarize the rating of the quality of evidence to make recommendations, the GRADE pro system was used for each outcome (https://gradepro.org/)^33^. Thus, we also presented the results using the summary of findings tables. In the subgroup analysis, two GRADE assessments were performed (one for each subgroup).

#### Clinical relevance

Assessment of clinical relevance was carried out using three categories: small effect (MD < 10% of the scale; SMD < 0.5); moderate effect (MD from 10 to 20% of the scale; SMD from 0.5 to 0.8); large effect (MD > 20% of the scale; SMD > 0.8)^34^.

## Results

**Figure 1:**
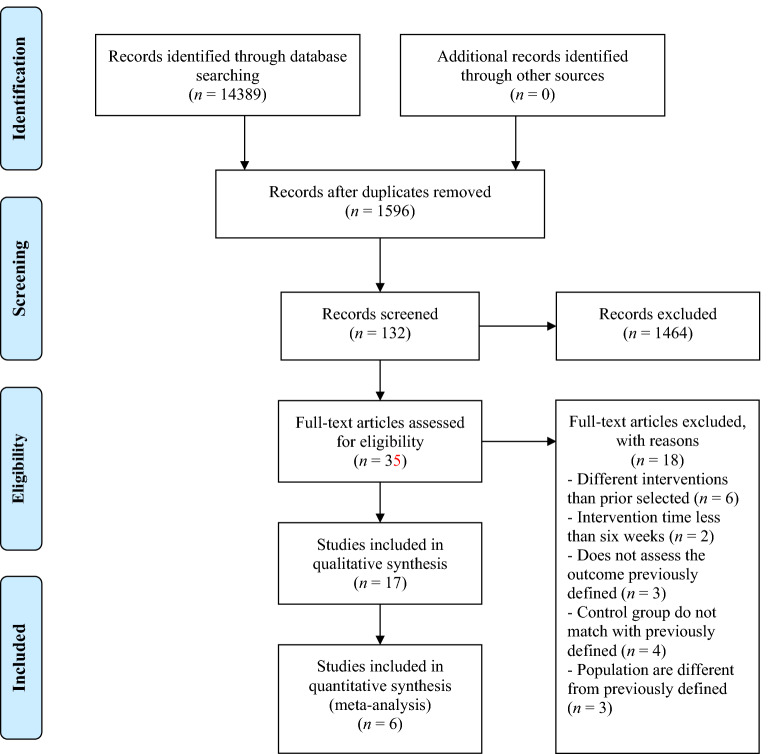
PRISMA flow diagram.

The electronic search retrieved 14,389 documents, of which 12,793 were excluded as duplicates, 1464 were excluded after screening by title and abstract, and 18 were excluded after full-text reading. Therefore, 17 studies^35–51^ were included in the qualitative synthesis after applying the eligibility criteria. Of these, six were included in the meta-analysis^35,37,41,46,48,51^. Figure 1 shows the search phases and screening of the studies included in the qualitative (systematic review) and quantitative (meta-analysis) synthesis.

### Characteristics of the included studies

The included studies had a total of 1117 participants with NSLBP of both sexes (254 of these included in meta-analysis procedures). The minimum and maximum ages of participants ranged between 18 and 55 years. The sample sizes of the included studies, considering all groups (experimental plus controls) ranged between 19 and 301 participants. The overall period of exercise interventions ranged from 6 to 16 weeks (10 [3.12]). The frequency of exercise interventions and management of control groups ranged from 1 to 5 times per week (3 [0.91]). The duration of exercise interventions (time of the session) ranged from 30 to 60 min. The intervention group exercises ranged from 1 to 18 exercises (4 [4.57]). The other characteristics of the included studies (intervention details, comparator, outcome measures, assessment, conclusion, methodological quality of clinical trials, and financial support) are presented in Table 1. The ongoing studies identified in the clinical trial database are presented in Table 2.

### Methodological quality assessment

Supplementary Table S2 shows that the PEDro score ranged from 3 to 8 points. Of the PEDro scale items, none of the studies scored on items 5 (blind subjects) and 6 (blind therapists). In contrast, all studies scored on item 10 (between group comparisons). Six studies^35,36,39–41,50^ were classified with high methodological quality (PEDro ≥ 6). The PEDro scale average score was 5.42.

### Studies not included in the meta-analysis and qualitative results

A total of 11 studies were excluded from the quantitative analysis: Maul (2005)^47^ did not report data from the control group; Smith (2011)^50^ reported only the effect size; França (2012)^40^ assessed a different outcome (abdominal musculature); Alp (2014)^39^ did not report the standard deviations; Macedo (2010)^45^ did not define LBP classifications; Rittwerger et al. (2002)^49^ could not be contacted for data availability; and Lomond (2015)^44^, You^43^ and Knox (2017)^42^ did not report data appropriately (the data were presented in graphs; strength values were adjusted using body weight; the data were presented in graphs, respectively); Bronfort et al.^36^ did not include a passive control; and Chok et al. (1999)^38^ had no study with which to compare the results. In addition, an email was sent to all authors, but Rittwerger et al. (2002)^49^ and Lomond et al.^44^ were unable to provide the data and the other authors did not respond to the email.

### Meta-analysis

The qualitative analysis shows that exercise interventions improve: (i) functional outcome^47^; (ii) strength of lumbar extensor muscles^50^; (iii) functional disability^40^; (iv) endurance^39^; (v) EMG outcome^44^; (vi) trunk muscle motor control^42^ and (vii) disability level^43^.

**Figure 2:**
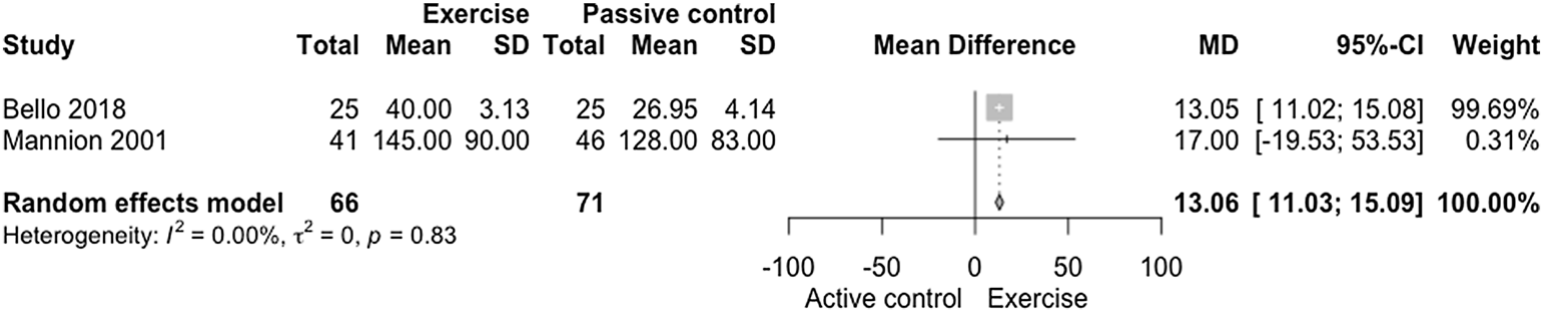
Forest plot of the comparison of exercise vs. active control on the EMG. Mannion et al. (2001)^41^: Training Devices versus Multimodal Physical therapy—Electromyogram (Hz). Bello (2018)^76^: Stabilization exercises versus Treadmill walking exercise—Electromyogram (Hz).

The meta-analysis on muscle electrical activity demonstrated a statistical difference for exercise interventions when compared to active control (Fig. 2; *n* = 137 participants; [experimental *n* = 66; control *n* = 71 participants], MD = 13.06 µV [11.03, 15.09], *p* < 0.0001), with low confidence in the effect estimate (Fig. 6, GRADE analysis of two studies). The clinical relevance found was small (Δ 8.42%). There was no heterogeneity in the muscle electrical activity analysis between exercise vs. active control on the EMG (I^2^ = 0%; *p* = 0.83).

**Figure 3:**
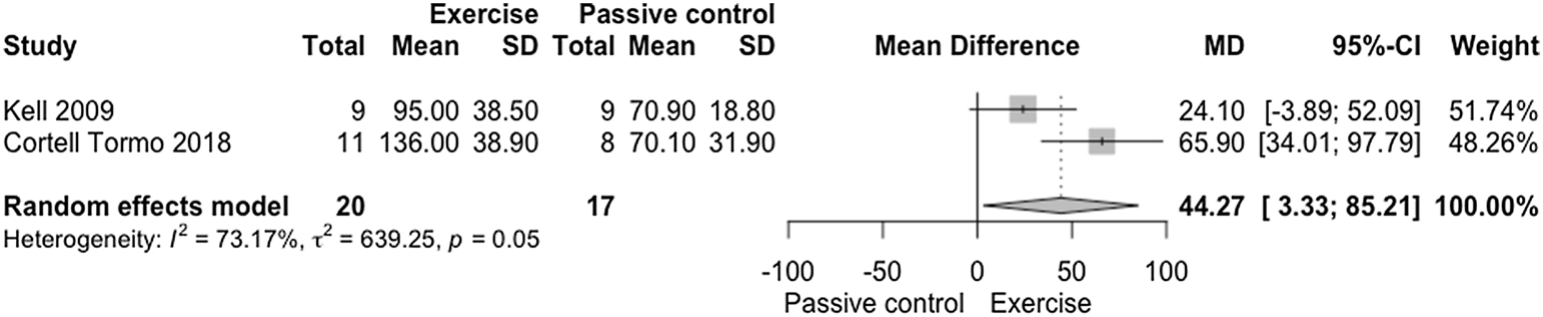
Forest plot of the comparison of exercise vs. passive control on trunk extensor endurance. Cortell-Tormo et al. (2018)^72^: Exercise versus No intervention—(s). Kell et al. (2009)^51^: Resistance exercises versus No intervention—Sorensen test (s).

The meta-analysis on muscle endurance of trunk extensors demonstrated statistical difference in favor of exercise interventions when compared to passive control (Fig. 3; *n* = 37 participants [experimental *n* = 20; control *n* = 17 participants], MD = 44.27 s [3.33, 85.21], *p* = 0.0340), with very low confidence in the effect estimate (Fig. 6, GRADE analysis of two studies). Large clinical relevance (Δ 31.39%) was found. There was substantial heterogeneity between exercise vs. passive control in the analysis of muscle endurance of trunk extensors (I^2^ = 73.17%; *p* = 0.05).

**Figure 4:**
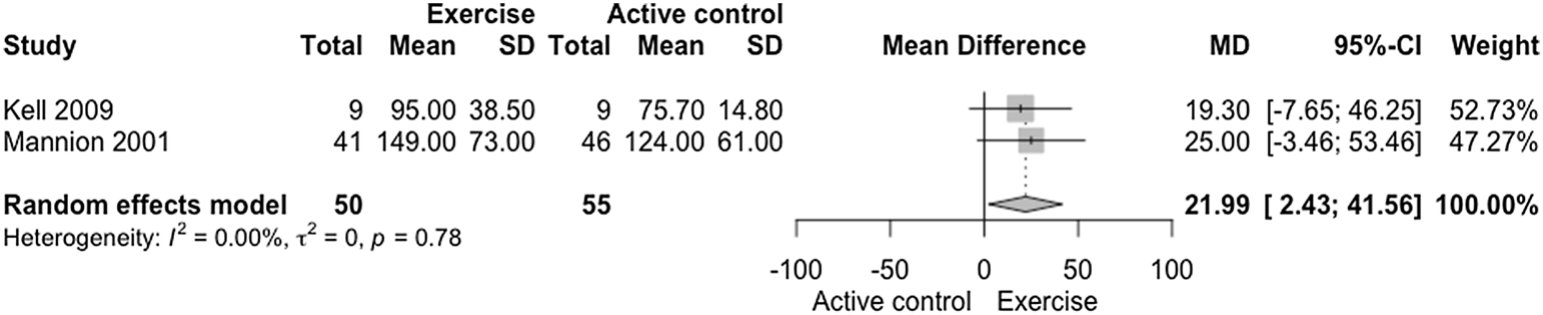
Forest plot of the comparison of exercise vs. active control on trunk extensor endurance. Mannion et al. (2001)^46^: Training Devices versus Multimodal Physical therapy; Sorensen test (seconds). Kell et al (2009)^51^: Resistance exercises versus Aerobic Training; Sorensen test (s).

The meta-analysis on trunk extensor muscle endurance demonstrated statistical difference in favor of exercise interventions when compared to active control (Fig. 4; *n* = 105 participants [experimental *n* = 50; control *n* = 55 participants], MD = 21.99 s [2.43, 41.56], *p* = 0.0276), with low confidence in the effect estimate (Fig. 6, GRADE analysis of two studies). Moderate clinical relevance (Δ 11.01%) was found. There was no heterogeneity between exercise vs. active control in muscle endurance of trunk extensors analysis (I^2^ = 0%; *p* = 0.78).

**Figure 5:**
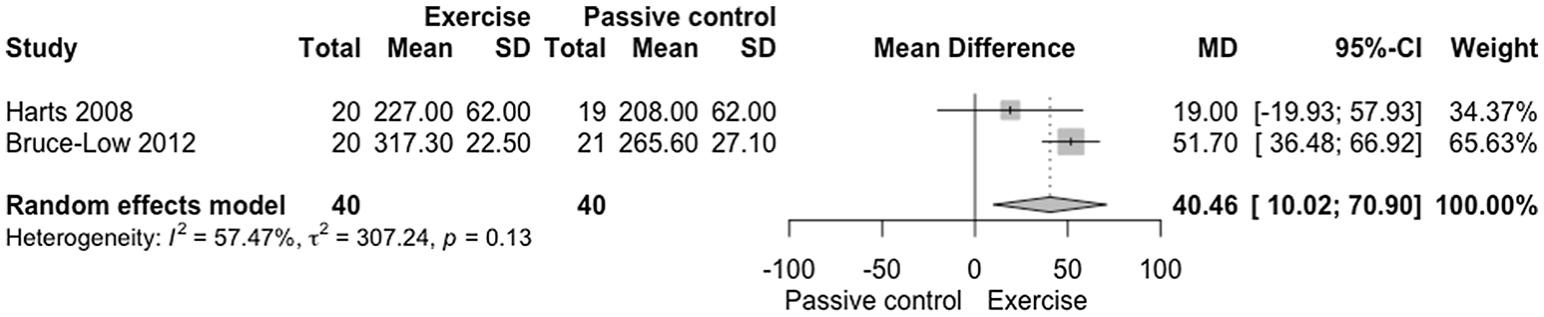
Forest plot of the comparison of exercise vs. passive control on trunk extensor strength. Harts (2008)^41^: Low-intensity training-LIT versus No intervention—Modified lower back machine (Newton meter). Bruce-Low et al. (2012)^37^: Exercise versus No intervention—Lumbar extension machine, Dynamometer (Newton meter).

**Figure 6:**
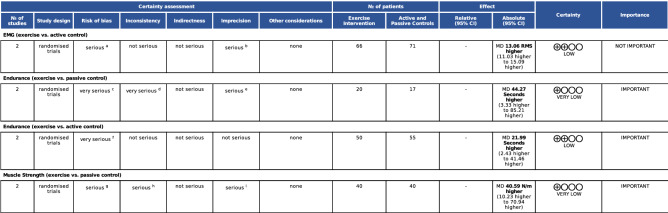
GRADE analysis. *CI* confidence interval; *MD* mean difference. Explanations: a = The study of Mannion et al. presented a Pedro score of 5/10 (low quality). b = Small effect (8.7%) requires a total sample size of approximately 400 (200 per group). c = The studies were classified as low quality (score 3/10 = Cortell-Tormo et al.^72^; score 4/10 = Kell et al.^51^). d = The heterogeneity between studies were substantial (I2 = 73%; *p* = 0.05). e = The confidence interval are very large (3.33–85.21 s). f = The studies were classified as low quality (score 5/10 = Mannion et al.^46^; score 4/10 = Kell et al.^51^). g = Bruce-low et al.^37^. The study does not provide concealed allocation and blinded assessments (score = 5/10). h = The heterogeneity between studies were reported by I2 statistics as 58% (substantial heterogeneity). i = The analysis performed are possible underpowered considering the Optimal Information Size (OIS) required for small clinical relevance (8.5%; under 10%). In this situation the OIS estimated are about 400 participants (200 per group).

The meta-analysis on muscle strength demonstrated statistical difference when compared to passive control (Fig. 5; *n* = 80 participants; [experimental *n* = 40; control *n* = 40 participants], MD = 40.46 N-meters [10.02, 70.90], *p* = 0.0092), with very low confidence in the effect estimate (Fig. 6, GRADE analysis of two studies]). The clinical relevance was small (Δ 8.54%). There was moderate heterogeneity in the analysis of muscle strength of trunk extensors (I^2^ = 57.47%, *p* = 0.13).

## Discussion

### Summary of main results

This systematic review compared exercise interventions with other types of exercise that are not explicitly designed to increase lumbar extensor muscle outcomes for people with NSLBP. Our quantitative analysis demonstrated that exercise interventions promote superior effects compared to active and passive controls for treating people with NSLBP. The superiority was demonstrated when exercise interventions were compared to: (i) active control on muscle electrical activity (small clinical relevance of 8.42%); (ii) passive control on muscle endurance (large clinical relevance of 31.39%); (iii) active control on muscle endurance (moderate clinical relevance of 11.01%); (iv) passive control on muscle strength (small clinical relevance of 8.54%).

The exercise interventions performed in the studies included in this systematic review are all classified as resistance training exercises. The literature review studies on this topic (not restricted to LBP) have presented exponential growth in recent decades, with more than 552 systematic reviews with meta-analysis published regarding resistance training exercises in the PubMed database. The classic outcomes in many of these reviews are changes in strength and hypertrophy, under different conditions, after manipulating acute and chronic training variables^52–57^. Resistance training is already recognized internationally as a medicine^58^, which is recommended in various conditions and diseases^59^. In the present review, we assessed three outcomes related to trunk extensor muscle function: strength, endurance, and myoelectrical activity.

The results with major clinical relevance were the effects of exercise interventions on muscle endurance when compared to passive control (large effect), with the control being less effective, although it is worth mentioning that few subjects were included in this analysis. There are multiple risk factors for developing back pain, including low back extensor endurance, and identifying these potential risks may be important in clinical practice^60^. Trunk extensor muscles are designed to support continuous activity throughout the day, but pain and inactivity alter these muscles so that they fatigue during activities of daily living^61^. The effects of exercise training in the present study were demonstrated by studies that used the Sorensen test, probably the most clinically useful test for clinical practice settings^62^. A previous study demonstrated that patients with chronic low back pain presented lower back extensor muscle isometric endurance than healthy subjects during the Sorensen test^63^. Here, the back muscle endurance outcome demonstrated that exercise interventions could be emphasized in rehabilitation strategies for subjects with chronic and subacute NSLBP. On the other hand, failure to exercise can increase general chronic pain^64^ and subacute LBP^65^.

The Sorensen test has also been used to analyze the trunk extensor fatigability based on the median frequency of electromyography analysis, and patients with LBP presented a significantly lower median EMG frequency in thoracic and lumbar regions, suggesting that individuals with low back pain demonstrated higher trunk fatigability^63,66^. Although in the present study the EMG of lumbar extensors demonstrated a small clinical effect, the results of the exercise intervention were superior to active control groups. Thus, the exercise interventions could also be indicated for subjects with NSLBP using some traditional modalities (multimodal physical therapy and treadmill walking exercise). Previous evidence from numerous studies demonstrated that lumbar extensors are active (EMG) during the performance of various exercises resulting from acute training^17^. Therefore, there is now some evidence of the potential of chronic adaptations using exercise training on machines for trunk extensors. Exercise interventions increase motor unit recruitment and firing rate^67^, and these alterations can increase muscle endurance^23^. In addition, resistance training increases EMG amplitude and muscle strength, suggesting a neural contribution^23^. Motor neurons, known as the final common pathway of neural activation signals, are improved by resistance training, leading to upregulation of agonist activity and possible intermuscular coordination of synergist muscles^68^. Likewise, resistance training can improve neural adaptations. Resistance exercise training also improves mitochondrial size and quantities^69^, which leads to an increase in ATP production^23^. Furthermore, mitochondria are responsible for lactate oxidization, which transforms lactate into glucose and provides body energy through the Cori cycle^23^. These statements show that resistance training can improve endurance, and people with NSLBP should use resistance training to improve trunk extensor muscle endurance.

Regarding muscle strength (exercise interventions vs. passive control), only a small clinical effect was demonstrated. These were surprising results because resistance training exercise has collectively been shown to be effective in increasing strength compared to non-exercise training-based treatments in adults^26^. Muscle weakness can lead to increased pain^70^ and decreased functionality^71^, and strength training is considered a treatment for these situations^72^. The dose–response to obtain gains from resistance training is a minimum of 4 sets per muscle group per week^73^. Neither of the studies used in the meta-analysis met this recommendation. One study^37^ performed only two sets per muscle group per week, and the other study^41^ performed only 1 set per muscle group per week. It is believed that the small clinical effect is due to not using the dose–response reported in the literature.

Other systematic reviews show that exercise interventions are effective and safe^15^ on subjective outcomes, such as reducing pain^13,14^, functional limitations^13,16^, disabilit^14^, and time to return to work^16^. In addition, strength training stimulates the release of serotonin and endorphins in the brain, which reduces pain and improves mood^74^. Therefore, our meta-analysis is in accordance with the positive results of previous systematic reviews^13–16^ that employed patient-centered outcomes (questionnaires). This means that exercise interventions also improve objective outcomes, such as muscle strength, muscle endurance, and electrical muscle activity. For practical and clinical application, exercise interventions, preferably resistance training, could be recommended for people with NSLBP.

This study has some limitations: First, the publication bias analysis was not employed considering the reduced number of articles included in the meta-analysis, such as analyzing the visual inspection (funnel plot) and the Egger test. These analyses require a minimum of 10 studies, according to the Cochrane Handbook. However, we performed a comprehensive search in many databases, and searches were also carried out in the gray literature and randomized clinical trial register databases. Second, despite the clinical effectiveness of exercise interventions, it should be noted that according to the GRADE analysis, there was no outcome with a moderate quality of evidence. The analyses show very low (muscle strength and endurance [passive control]) and low (electromyography and endurance [active control]) quality evidence that exercise interventions are effective when compared to the control groups investigated. Third, the influence of variables related to the exercise prescription (duration, frequency, number of repetitions, intensity, movement speed, and rest interval)^75^ was not considered in the meta-analytical procedures. Although this influence can be analyzed by the meta-regression approach, unfortunately, the analysis could not be performed with only two studies. Fourth, we cluster all interventions, even with different exercises, despite the fact that there are different demands on physical capabilities for each exercise. Fifth, the instruments for assessing strength and endurance are different between the studies included in the meta-analysis. This is a common situation when combining studies for meta-analytical procedures. However, we standardized the measurement units to use the mean difference summary effect as a statistical approach, in order to provide clinical applicability to the results. Finally, although the meta-analysis procedures were performed with two studies for all outcomes, it was decided to maintain the meta-analytical^22^ results to provide absolute values that could be extrapolated for health professional use. Future systematic reviews with meta-analysis are needed using studies with high confidence and filling the remaining gaps.

## Conclusion

Our study demonstrates that chronic exercise interventions (more than 6 weeks) can be effective in adults with NSLBP and should be incorporated into clinical practice to promote muscle adaptations. There are few studies included in the meta-analysis (only 2 per outcome), and therefore the results should be taken with precaution. From the GRADE analysis, almost all included studies were of low-quality confidence, also the results show small clinical evidence for several of the outcomes. There was very low-quality evidence that exercise interventions were effective to increase muscle strength and endurance when compared to passive control (no intervention). There was low quality evidence that exercise interventions were effective to increase muscle endurance and myoelectrical activity when compared to active control (multimodal physical therapy, aerobic training, and treadmill walking exercise).

## Supplementary Information

Supplementary Table 1.

Supplementary Table 2.
