# Supplementary material for: Exercise interventions can improve muscle strength, endurance, and electrical activity of lumbar extensors in individuals with non-specific low back pain: a systematic review with meta-analysis
Source: Sci Rep. 2021 Aug 19;11:16842. doi: 10.1038/s41598-021-96403-7 (PMC8376921; doi:10.1038/s41598-021-96403-7)
Supplement: Supplementary file 1 — Supplementary Table 1. [file 41598_2021_96403_MOESM1_ESM.docx]

**Supplementary Table 01 -** General search strategy made in July until August, 2020.

| **Search** | **Query** | **No. Of item found** |
| --- | --- | --- |
| #1 | (((((((((((((((((((((exercises) OR (exercise)) OR (physical activities)) OR (physical activity)) OR (remedial exercise)) OR (remedial exercises)) OR (Exercise Therapies)) OR (Rehabilitation Exercise)) OR (Rehabilitation Exercises)) OR (Exercise Movement Technics)) OR (Strength Training)) OR (Resistance Training)) OR (Muscle Strength)) OR (Muscle Strengthening)) OR (Strengthening Exercise)) OR (Physical Exercise)) OR (Circuit-Based Exercise)) OR (Exercise Movement Techniques)) OR (Exercise Therapy)) OR (High-Intensity Interval Training)) OR (Endurance training)) AND (Randomized Controlled Trial[Publication Type]) | 50,725 |
| #2 | ((((((((((((Low Back Pain) OR (Low Back Pains)) OR (Lumbago)) OR (Lower Back Pain)) OR (Lower Back Pains)) OR (Low Back Ache)) OR (Low Back Aches)) OR (Low Backache)) OR (Low Backaches)) OR (Postural Low Back Pain)) OR (Recurrent Low Back Pain)) OR (Mechanical Low Back Pain)) AND (Randomized Controlled Trial[Publication Type]) | 3,424 |
| Final | #1 AND #2 | 3,141 |
