# Supplementary material for: Exercise interventions can improve muscle strength, endurance, and electrical activity of lumbar extensors in individuals with non-specific low back pain: a systematic review with meta-analysis
Source: Sci Rep. 2021 Aug 19;11:16842. doi: 10.1038/s41598-021-96403-7 (PMC8376921; doi:10.1038/s41598-021-96403-7)
Supplement: Supplementary file 2 — Supplementary Table 2. [file 41598_2021_96403_MOESM2_ESM.docx]

**Supplementary Table 02**  – PEDro scale.

| **Year, title** | **1**  **Eligibility criteria** | **2**  **Random allocation** | **3**  **Concealed allocation** | **4**  **Baseline Comparability** | **5**  **Blind subjects** | **6**  **Blind therapists** | **7**  **Blind assessor** | **8**  **< 15% of dropouts** | **9**  **Intention to treat analysis** | **10**  **Between groups comparison** | **11**  **Point estimates and variability** | **Total** |
| --- | --- | --- | --- | --- | --- | --- | --- | --- | --- | --- | --- | --- |
| Chok, 1999* | 1 | 1 | 0 | 1 | 0 | 0 | 0 | 0 | 0 | 1 | 1 | 4 |
| Mannion, 2001* | 1 | 1 | 0 | 1 | 0 | 0 | 0 | 1 | 0 | 1 | 1 | 5 |
| Rittweger, 2002* | 1 | 1 | 0 | 1 | 0 | 0 | 0 | 1 | 0 | 1 | 1 | 5 |
| Maul, 2005 | 1 | 1 | 0 | 1 | 0 | 0 | 0 | 0 | 0 | 1 | 1 | 4 |
| Harts, 2008* | 1 | 1 | 1 | 1 | 0 | 0 | 1 | 1 | 1 | 1 | 1 | 8 |
| Kell, 2009* | 1 | 1 | 0 | 1 | 0 | 0 | 0 | 0 | 0 | 1 | 1 | 4 |
| Macedo, 2010* | 0 | 1 | 1 | 0 | 0 | 0 | 1 | 0 | 0 | 1 | 1 | 5 |
| Bronfort, 2011* | 1 | 1 | 1 | 1 | 0 | 0 | 1 | 1 | 1 | 1 | 1 | 8 |
| Smith, 2011 | 1 | 1 | 0 | 1 | 0 | 0 | 0 | 1 | 1 | 1 | 1 | 6 |
| França, 2012 | 1 | 1 | 1 | 1 | 0 | 0 | 1 | 1 | 1 | 1 | 1 | 8 |
| Bruce-Low, 2012 | 0 | 1 | 0 | 1 | 0 | 0 | 0 | 1 | 0 | 1 | 1 | 5 |
| Alp, 2014 | 1 | 1 | 0 | 1 | 0 | 0 | 1 | 1 | 0 | 1 | 1 | 6 |
| You, 2015 | 0 | 1 | 0 | 1 | 0 | 0 | 0 | 0 | 0 | 1 | 1 | 4 |
| Lomond, 2015 | 1 | 1 | 0 | 1 | 0 | 0 | 1 | 1 | 0 | 1 | 0 | 5 |
| Knox, 2017 | 1 | 0 | 0 | 1 | 0 | 0 | 1 | 1 | 0 | 1 | 1 | 5 |
| Cortell-Tormo, 2018* | 1 | 1 | 0 | 0 | 0 | 0 | 0 | 0 | 0 | 1 | 1 | 3 |
| Bello, 2018* | 1 | 1 | 1 | 1 | 0 | 0 | 1 | 1 | 1 | 1 | 1 | 8 |

* = Studies included in the meta-analysis.
